# Erratum: A novel humanized mouse lacking murine p450 oxidoreductase for studying human drug metabolism

**DOI:** 10.1038/s41467-017-01314-9

**Published:** 2017-10-17

**Authors:** Mercedes Barzi, Francis P. Pankowicz, Barry Zorman, Xing Liu, Xavier Legras, Diane Yang, Malgorzata Borowiak, Beatrice Bissig-Choisat, Pavel Sumazin, Feng Li, Karl-Dimiter Bissig

**Affiliations:** 1 Center for Cell and Gene Therapy, Stem Cells and Regenerative Medicine Center, Baylor College of Medicine, Houston, TX 77030 USA; 2 Graduate Program, Department of Molecular and Cellular Biology, Baylor College of Medicine, Houston, TX 77030 USA; 3 Texas Children’s Cancer Center, Department of Pediatrics, Baylor College of Medicine, Houston, TX 77030 USA; 4 Alkek Center for Molecular Discovery, Advanced Technology Core, Baylor College of Medicine, Houston, TX 77030 USA; 5 Department of Molecular and Cellular Biology, Baylor College of Medicine, Houston, TX 77030 USA; 6 Program in Developmental Biology, Baylor College of Medicine, Houston, TX 77030 USA; 7 Dan L. Duncan Cancer Center, Baylor College of Medicine, Houston, TX 77030 USA; 8 McNair Medical Institute, Houston, TX 77030 USA

*Nature Communications* **8**:39 doi:10.1038/s41467-017-00049-x; Article pubilshed online: 28 June 2017

An incorrect version of the Supplementary Information was inadvertently published with this article where the wrong file was included. The HTML has been updated to include the correct version of the Supplementary Information.

