# Supplementary material for: A novel humanized mouse lacking murine P450 oxidoreductase for studying human drug metabolism
Source: Nat Commun. 2017 Jun 28;8:39. doi: 10.1038/s41467-017-00049-x (PMC5489481; doi:10.1038/s41467-017-00049-x)
Supplement: Supplementary file 1 — Supplementary Information [file 41467_2017_49_MOESM1_ESM.pdf]

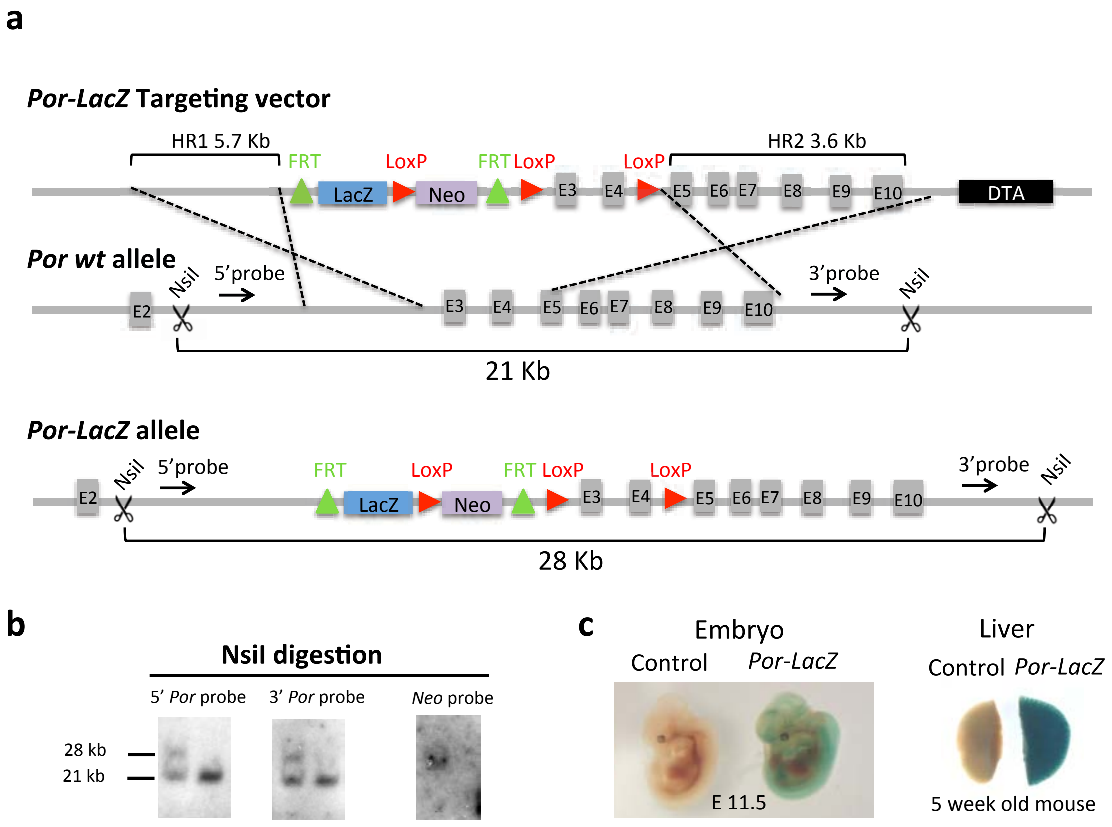

Supplementary Figure 1 | Vector design and targeting of the *Por* gene (a) design of targeting vector and modified *Por* locus. (**b**) Southern blotting with three probes indicates proper targeting of ESC (first lane) and wild-type control ESC (second lane) (**c**) Beta galactosidase (*lacZ*) expression from the *Por-lacZ* allele. X-gal staining of heterozygous mouse embryo and liver demonstrating expression of the galactosidase from the *Por-lacZ* allele.

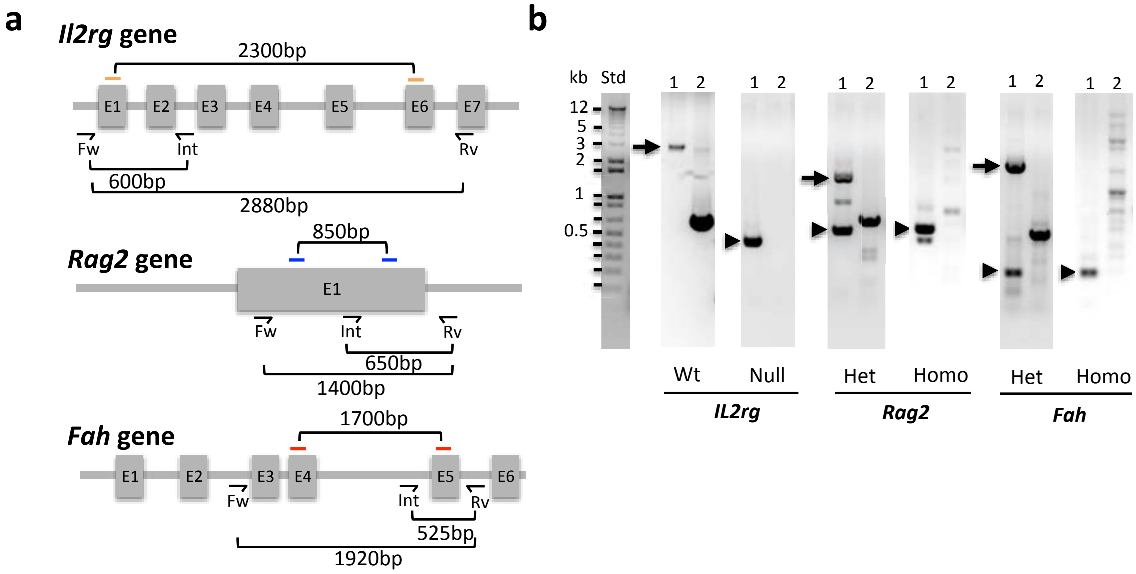

Supplementary Figure 2 | Detection of genomic deletions by size of PCR products of PIRF founder mice. (**a**) Scheme of *Il2rg*, *Rag2* and *Fah* genes showing the gRNA location and the primers used to genotype the mice and the expected size of the wild type band. **(b)** DNA agarose gel pictures of the PCRs obtained from genotyping. Lane 1, PCR bands using external (Fw and Rev) primers from the gRNA sites. For *Rag2* and *Fah* heterozygotes, a wild type (arrow) and a deleted (arrowhead) band could be detected. *Il2rg* is a X-linked gene and no founder heterozygote females were generated. Null males for *Il2rg* and homozygotes for *Rag2* and *Fah* showed a single deleted genotyping band of 460bp, 530bp and 200bp, respectively (according with the deletion size of supplementary figure 3 table). Lane2, PCR bands using one of the external (Fw or Rev) and the internal (Int) primer located between the two gRNA sites.

a

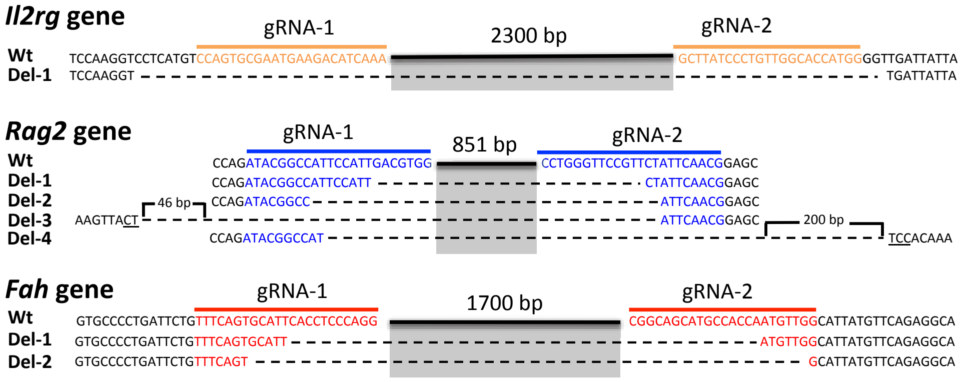

b

Gene deletion	size (bp)	Protein modification
Il2rg$^{-/-}$ **Del-1**	2422bp	Frame shift from aminoacid 28:<br>LCWHYVQRQGECAVAKLAPLTCGIPWPSFLHCGIWNPDSKTHGADET+STOP
Rag2$^{-/-}$ **Del-1**	871bp	Frame shift from aminoacid 143:<br>ILFNGAQ+STOP
Rag2$^{-/-}$ **Del-2**	877bp	Frame shift from aminoacid 141:<br>IQRSSINPP+STOP
Rag2$^{-/-}$ **Del-3**	936bp	Frame shift from aminoacid 122:<br>IQRSSINPP+STOP
Rag2$^{-/-}$ **Del-4**	1088bp	Frame shift from aminoacid 142:<br>TKKALGKS+STOP
Fah$^{-/-}$ **Del-1**	1717bp	Frame shift from aminoacid 107:<br>LCWHYVQRQGECAVAKLAPLTCGIPWPSFLHCGIWNPDSKTHGADET+STOP
Fah$^{-/-}$ **Del-2**	1720bp	Frame shift from aminoacid 107:<br>LCSEARRMRCCQIGSTYLWDTMAELPPLWLEPRFEDPWGR+STOP

Supplementary Figure 3 | Spectrum of genomic deletions in the *Il2rg*, *Rag2* and *Fah* genes. CRISPR/Cas9 injected zygotes of conditional *Por*$^{c/c}$ mice were sequenced to determine deletion on DNA (**a**) and amino-acid level (**b**).

a

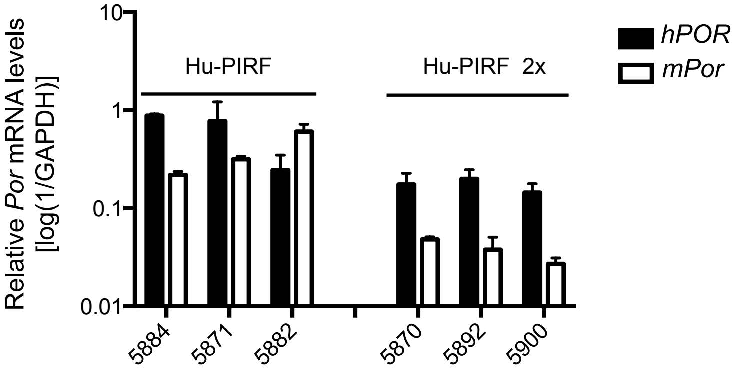

b

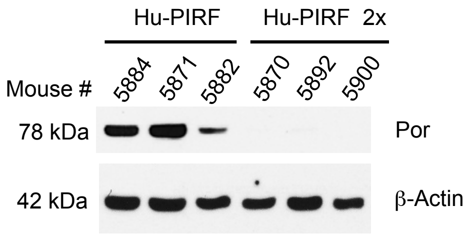

Supplementary Figure 4 | Expression of P450 oxidoreductase in humanized PIRF mice. Humanized PIRF mice were injected with adenovirus expressing CRE before and after transplantation. Livers harvested seven days after the second injection for expression analysis (**a**) qPCR of human and murine specific *Por* normalized to human and murine *Gapdh,* respectively. (**b**) Western blotting for murine Por and β-actin of liver samples from the same humanized mice. Results are expressed in mean values ± SEM of triplicates of the same sample. PIRF; *Por*$^{c/c}$*/Il2rg*$^{-/-}$*/Rag2*$^{-/-}$*/Fah*$^{-/-}$.

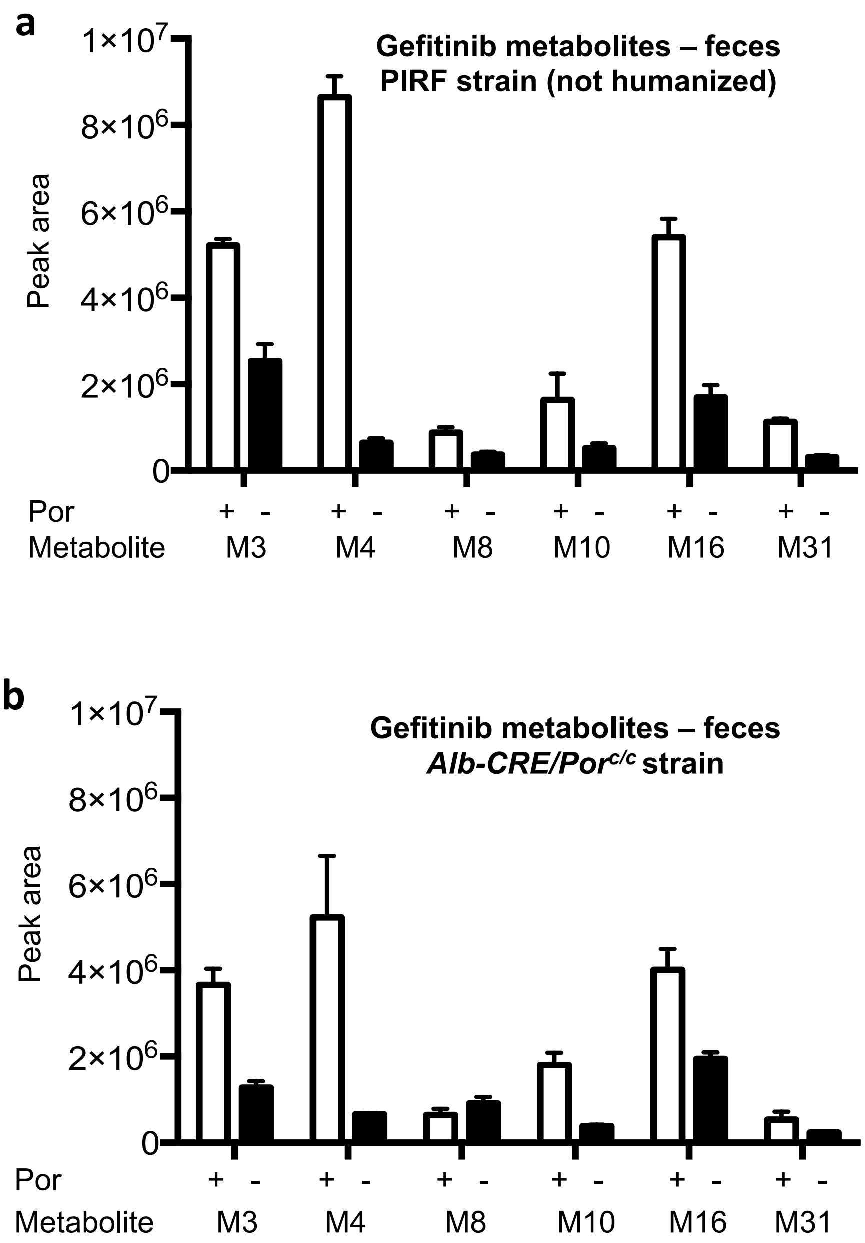

Supplementary Figure 5 | Gefitinib metabolites upon murine P450 oxidoreductase (*Por*) deletion. (a) Deletion by adenoviral delivery of CRE, (**b**) deletion by crossing of $Por^{c/c}$ with *Alb-Cre* mice, generating an *Alb-Cre*/*Por*$^{c/c}$ strain. Peak area for selected gefitinib metabolites in feces are given. Results are expressed in mean values ± SEM of three mice (N=3). PIRF; *Por*$^{c/c}$/*Il2rg*$^{-/-}$/*Rag2*$^{-/-}$/*Fah*$^{-/-}$.

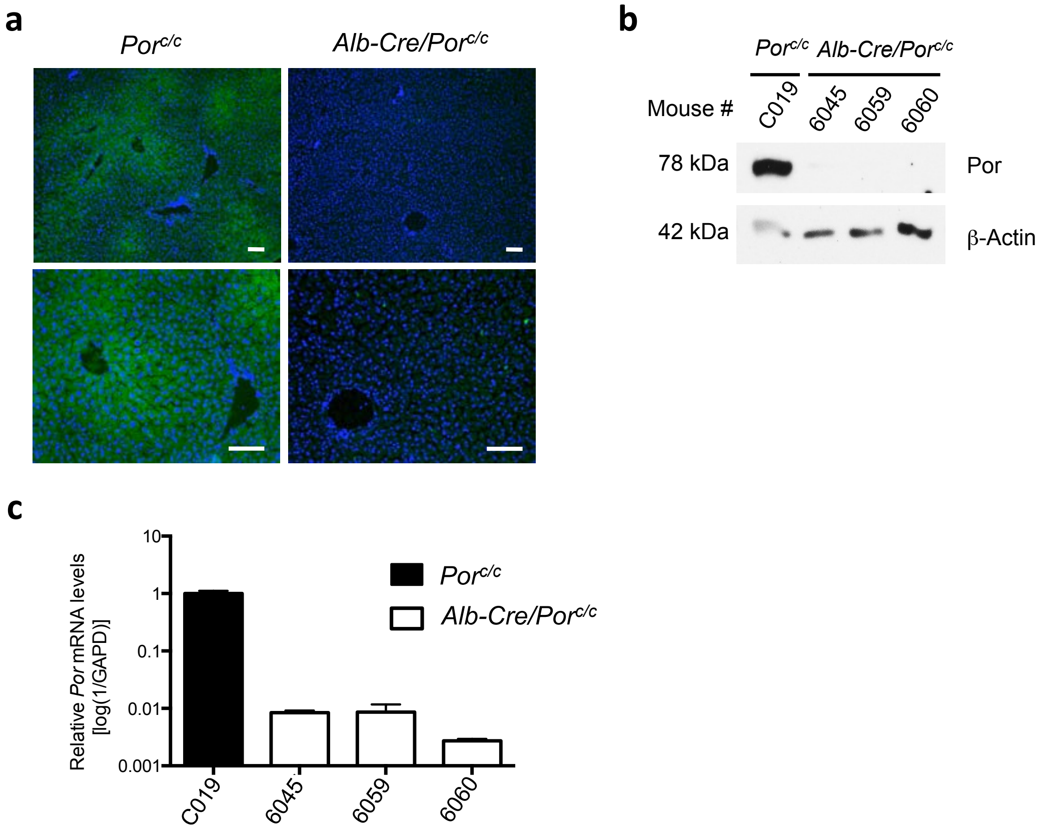

Supplementary Figure 6 | Deletion of murine P450 oxidoreductase by crossing *Por*$^{c/c}$ with an *Alb-Cre* transgenic mouse. Homozygous *Por*$^{c/c}$ mice were crossed with a transgenic strain expressing CRE recombinase from the albumin promoter. *Alb-CRE/Por*$^{c/c}$ mice were harvested after 4 weeks and analyzed. (**a**) Representative immunostaining showing virtually complete Por deletion (**b**) Western blotting for Por protein using liver samples from a control *Por*$^{c/c}$ mouse and three different *Alb-Cre/Por*$^{c/c}$ mice (**c**) qPCR showing murine *Por* mRNA levels of the same mice. Results are expressed in mean values ± SEM of triplicates of the same sample. Scale bar 50 μm.

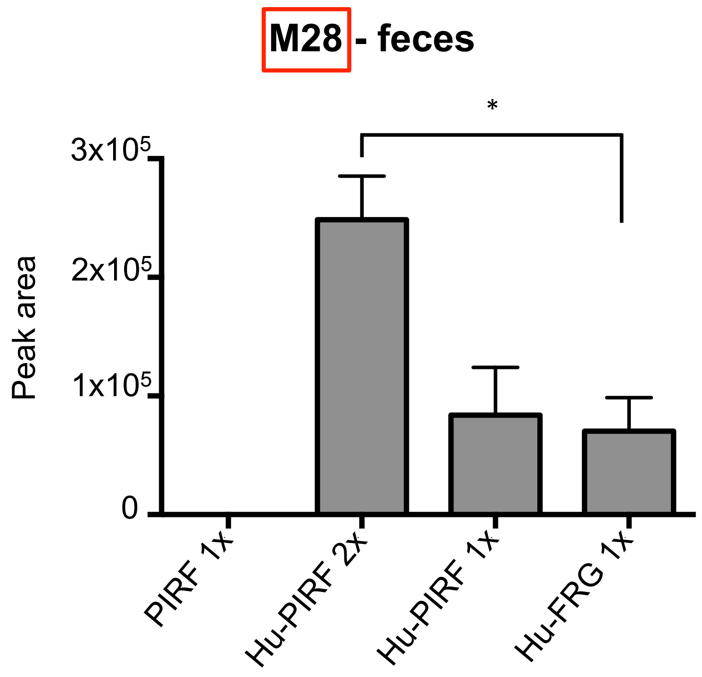

Supplementary Figure 7 | Gefitinib metabolite M28 in feces. Peak area of M28 in feces from different humanized mice. Same data as Fig. 4d but expressed in absolute values for different humanized and control PIRF mice. Results are expressed in mean values ± SEM of 3 mice (N=3). * $p < 0.05$ using Mann-Whitney test.
PIRF; *Por*$^{c/c}$/*Il2rg*$^{-/-}$/*Rag2*$^{-/-}$/*Fah*$^{-/-}$, FRG; *Fah*$^{-/-}$/*Rag2*$^{-/-}$/*Il2rg*$^{-/-}$.

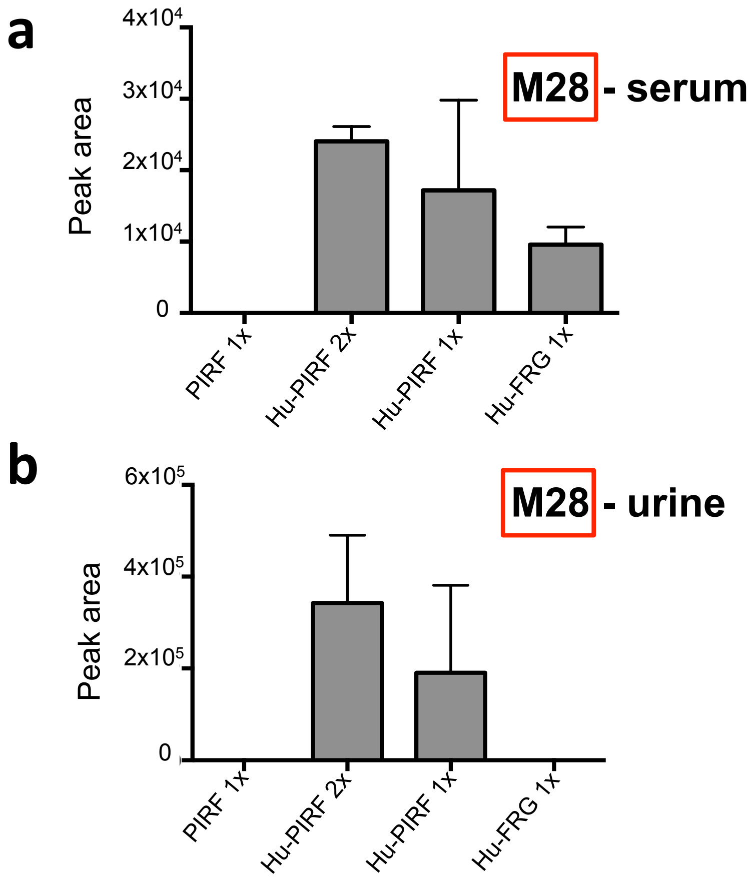

Supplementary Figure 8 | Gefitinib metabolite M28 in the serum and urine of hu-PIRF-2x mice and control groups. Peak area in serum (**a**) and urine (**b**) is given. Results are expressed in mean values ± SEM of 3 mice (N=3).
PIRF; $Por^{c/c}/Il2rg^{-/-}/Rag2^{-/-}/Fah^{-/-}$, FRG; $Fah^{-/-}/Rag2^{-/-}/Il2rg^{-/-}$.

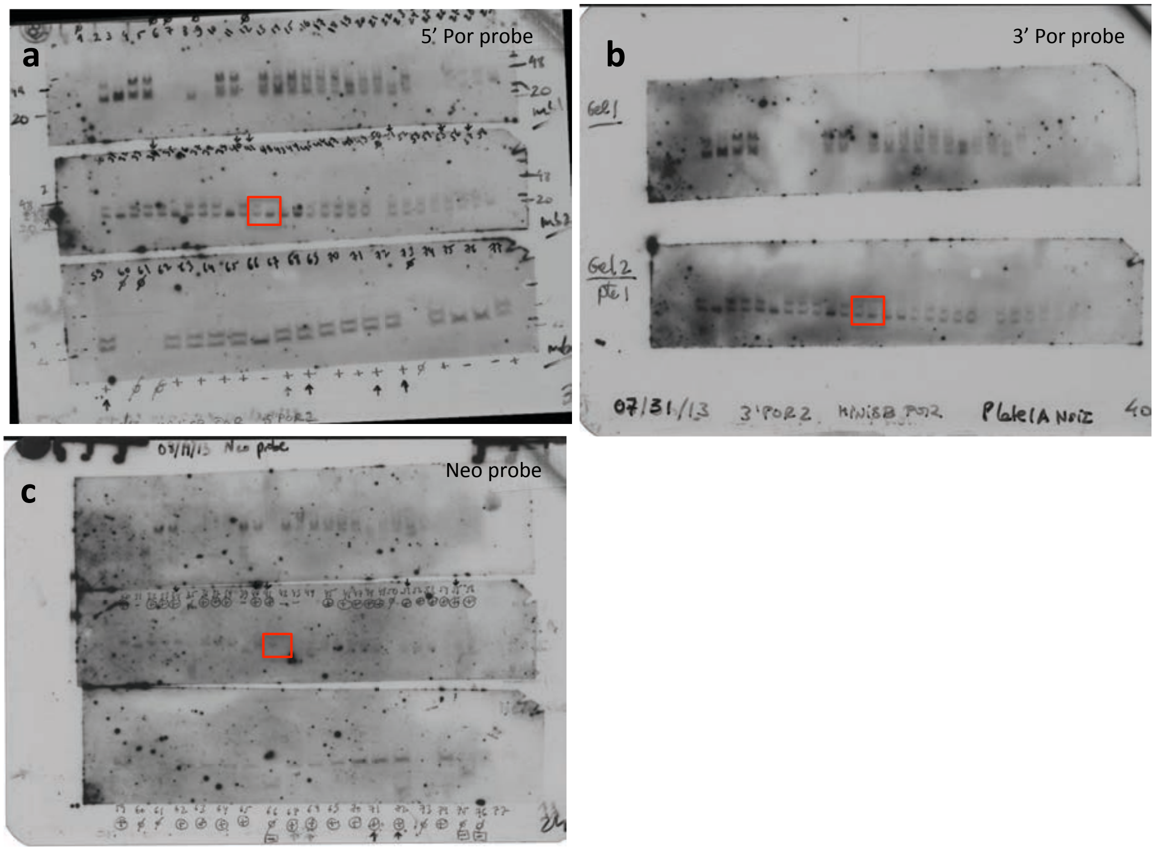

Supplementary Figure 9 | Full images of targeted ESCs Southern blots. The same blots as Supplementary Fig. 1b using a 5'probe (a) a 3' probe (b) and neomycin probe (c).

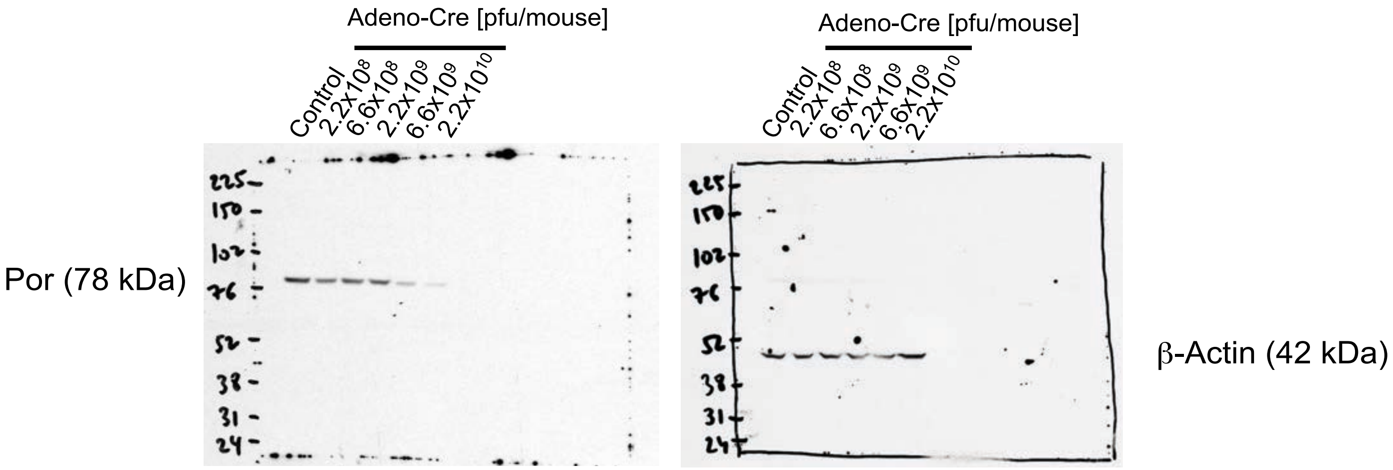

Supplementary Figure 10 | Full images of murine Por and Gadph Western blots from PIRF mice injected with Adeno-CRE. The same blots as Fig. 1.

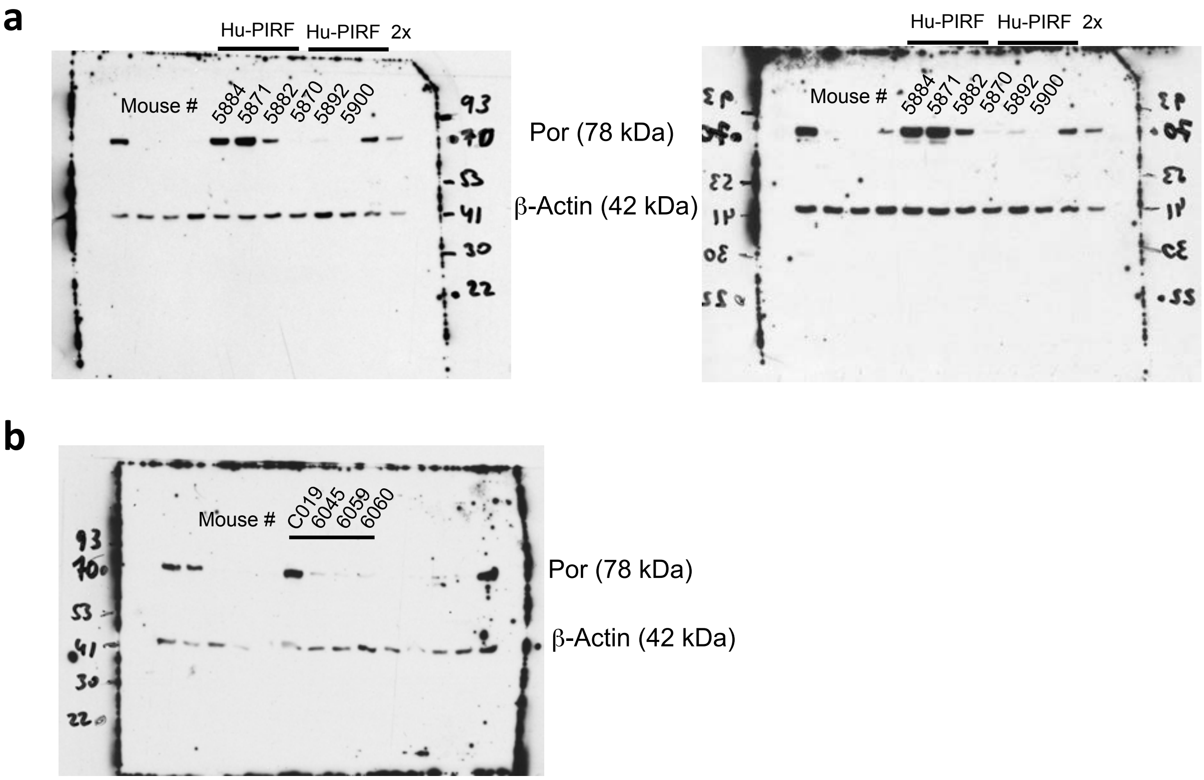

Supplementary Figure 11 | Full images of murine Por and Gadph Western blots. The same blots as Supplementary Fig. 4 and 6. Humanized PIRF mice (a) and *Porc/c* and *Porc/c/Alb-CRE* mice (b).

Hepatocyte	Age*	Gender	Race	BMI	Cause of death	Usage in present study
#1	24	Male	African American	20.3	Anoxia	RNAseq (chimeric mice, hepatocytes)
#2	2	Female	African American	19.6	Head trauma	RNAseq (chimeric mice, hepatocytes)
#3	45	Female	Caucasian	20.8	Anoxia	RNAseq (chimeric mice)
#4	1.2	Female	Caucasian	20.8	Head trauma	Gefetinib metabolites
#5	18	Male	Caucasian	24.3	Cardiovascular	ATV metabolites

* in years

Supplementary Table 1 | Characteristics of human hepatocyte donors used in present study.

**Murine P450 cytochromes**	**Present work**	**Weng et al. 2005**
	Change fold (*Por*-deleted/*Por* non-deleted mice)	
Cyp2a4	1.4	4.5
Cyp2a5	1.3	4.5
Cyp2b10	12.4	15.8/16.3/9.1
Cyp2c39	1.8	1.4
Cyp2c55	14.6	17.2
Cyp4a10	3.7	0.3/0.7
Cyp7a1	4.6	3.1/4.9
Cyp7b1	1.6	0.2/0.3
Cyp26a1	6.7	3.5
Cyp51	0.6	2.2

Supplementary Table 2 | Comparison of murine gene expression profiles of chimeric livers to previously published non-humanized mice. Gene expression of conditional (Alb-Cre) *Por* KO mice have been quantified by microarray analysis (Weng et al. 2005 *J Biol Chem* **280**, 31686-31698 (2005)). Present study used RNA-Seq to compare the gene expression (Fig. 2b) in humanized livers transduced with Adeno-Cre and Adeno-GFP. This table lists all previously published cytochromes with values (fold changes) compared to our data set. Multiple numbers represent multiple sets of microarray probes.
